# A TOPSIS-Inspired Ranking Method Using Constrained Crowd Opinions for Urban Planning

**DOI:** 10.3390/e24030371

**Published:** 2022-03-05

**Authors:** Sujoy Chatterjee, Sunghoon Lim

**Affiliations:** 1 Informatics Cluster, School of Computer Science, University of Petroleum and Energy Studies (UPES), Dehradun 248007, India; sujoy.chatterjee@ddn.upes.ac.in; 2 Department of Industrial Engineering, Ulsan National Institute of Science and Technology, Ulsan 44919, Korea; 3 Institute for the 4th Industrial Revolution, Ulsan National Institute of Science and Technology, Ulsan 44919, Korea

**Keywords:** crowdsourcing, decision making, multi-attribute decision problems, urban planning

## Abstract

Crowdsourcing has become an important tool for gathering knowledge for urban planning problems. The questions posted to the crowd for urban planning problems are quite different from the traditional crowdsourcing models. Unlike the traditional crowdsourcing models, due to the constraints among the multiple components (e.g., multiple locations of facilities) in a single question and non-availability of the defined option sets, aggregating of multiple diverse opinions that satisfy the constraints as well as finding the ranking of the crowd workers becomes challenging. Moreover, owing to the presence of the conflicting nature of features, the traditional ranking methods such as the Technique for Order of Preference by Similarity to Ideal Solution (TOPSIS) cannot always be feasible as the optimal solutions in terms of multiple objectives cannot occur simultaneously for the conflicting cases (e.g., benefit and cost criteria) for urban planning problems. Therefore, in this work, a multi-objective approach is proposed to produce better compromised solutions in terms of conflicting features from the general crowd. In addition, the solutions are employed to obtain a proper ideal solution for ranking the crowd. The experimental results are validated using two constrained crowd opinion datasets for real-world urban planning problems and compared with the state-of-the-art TOPSIS models.

## 1. Introduction

Crowd-powered systems [1,2] have already been adopted as a powerful tool for resolving a complex task in a distributed manner within a limited time and a feasible budget. It is noticed that proper deployment of enormous human resources to solve a critical task can be beneficial even as the alternative of hiring quality experts. Despite the numerous advantages of employing crowd opinions, there exists a spectrum of challenges with a high possibility of spamming. The reason is due to the abrupt behaviour of crowds, because they can malfunction the overall process.

In various real-life problems, such as urban planning, it is always required to assemble diverse public preferences, in order to realize the practical need of some particular resources in a locality. Hence, crowdsourced opinions have an important impact on the decision making to the professional planner [3,4,5]. During the last couple of years, posting a question to crowd and seeking their opinions in an online manner for some specific questions are demonstrated to be very fruitful [6,7,8]. However, on account of the fact that there exists numerous uncertainty in the behaviour of the crowd workers, hence, a proper aggregation of their opinions and ranking of them are difficult problems. With the passage of time, for different tasks, a spectrum of the methods have been proposed to aggregate multiple crowd opinions to derive proper judgment [6,7,8,9,10,11,12,13,14]. A majority of research available in the literature deals with the crowd judgment problem, where the opinions are basically either binary or multiple option types. Literally, this denotes that the option sets are defined and one particular opinion is chosen from the opinion set. However, in our day-to-day life, there are some problems (e.g., urban planning problems), where there is no predefined option set available [15]. Thus, it makes the problem really challenging to obtain an aggregated judgment from the original crowd opinions and making a ranking among the crowd workers. Moreover, most of the state-of-the-art research deals with the problem of judgment analysis considering the crowd opinions as either binary (’Yes’ and ’No’) or multiple (’Yes’, ’No’, ’Skip’, ’I can’t tell’, etc.) option set. A previous study dealt with the recently introduced new type of judgment analysis model, termed ’constrained judgment analysis’ [11], where the defined option set is not available. In addition to that, ranking of crowd is very necessary to motivate them for next opinion collections. However, ranking of the crowd becomes more challenging in this constrained judgment setting, as there is no defined options set. Thus, the motivation of this paper is two fold. The first one is to propose a multi-objective algorithm to simultaneously optimize two conflicting objective functions. It aims to directly compute the improved opinions from the constrained crowdsourced opinions without binning. Then, the obtained improved solution is employed to define a proper ideal solution in order to find better ranking of the crowd workers.

In the traditional judgment analysis problems [6,16], while posting a question to seek the public opinions, the predefined option sets are also mentioned. From this predefined option set, people can choose the appropriate option according to their viewpoints. But in many different real-life problems (e.g., urban planning problems), the option sets are not defined always, rather only the ranges are available. To explain this with an example of urban planning, suppose one agency wishes to install *k* ATM counters in a locality, but there can be some locations where people feel some hindrances due to the geographical nature. Thus, gathering public opinions through outsourcing is found to be an alternative choice to the decision makers. Here, each *k* opinion is basically a 2D value and each of the location can be thought of as a component. Accurate planning requires that there should be some specific distances between the pair of ATM counters. Hence, there should be a minimum distance (i.e., a constraint) between any two ATM counters and the crowd workers should satisfy the constraint.

In the traditional crowdsourcing market [16,17,18], the posted question should have only one component and there is no sub-component in it. However, in this example, the question is comprised of some sub-components (i.e., *k* components referring *k* locations) and this evolves a new avenue of research to find a proper aggregation from multiple crowd opinions. Here, as there are *K* locations so *K* number of 2D values should be present there. In this type of problems, there is no defined option set rather only the range of a coordinate value is available by demonstrating a grid map of a particular locality. In the traditional judgment analysis model, as the option sets are defined, therefore, different opinion aggregation models including majority voting can be applied and ranking can be easily obtained by their accuracy. However, these models are not applicable for this type of constrained judgment analysis, because there is no predefined option set and a much smaller chance that two crowd workers’ have the same opinions. Thus, an application of even majority voting is less feasible here. On the other hand, different probabilistic models require us to find the posterior distribution of each option and based on that the decision can be made. Nevertheless, these types of procedures are also not applicable in this scenario.

Next, in order to motivate the crowd workers to provide the high-quality opinion, a rank-based sorting depending upon their expertise among the crowd workers is very necessary. Based on the ranking, the decision makers can have the choice of selecting the solution according to their needs. In addition to that, in order to motivate the crowd workers for providing their opinions, a reward scheme can be interesting, thus the ranking of crowd workers is very helpful for that purpose. To provide a good ranking from the constrained opinion of the crowd workers, we need to first extract the feature values of the opinions based on the choices of the decision makers. In this example, the two objective functions are defined as (1) a coverage area pointed out by the *k* locations and (2) a deviation of a particular crowd opinion from the mean opinions. The deviation is taken into account in order to prevent the outlier solution. However, in this kind of problems, no defined weight about the two features is available from the decision makers, rather only the priority is available. In this example, suppose that the first objective (i.e., the coverage) is considered to have a high priority to the decision makers. The reason is that any solution with zero deviation and very low coverage cannot be considered as a good one due to its inability to distribute the resources among vast people. Rather, a good compromise i.e., highest value in coverage and lower value in deviation (not necessarily lowest) between the two objectives can be treated as the good one. Interestingly, in order to produce the proper ranking, the traditional group decision making model, that is, the Technique for Order of Preference by Similarity to Ideal Solution (TOPSIS) [19] can be applied there. During the last couple of years, numerous versions of the TOPSIS models came into play in order to resolve different issues related to weight criteria, normalization issues, and so forth. However, in these settings, determining a proper ideal solution is crucial, as the ranking can be changed due to the chosen ideal solution and the normalization procedure. Recently, three weighted TOPSIS models are introduced that showed their efficiencies over the existing approaches in the domain of lean construction and wireless sensor networks [20,21]. In these approaches, some novel techniques using entropy and standard deviation-based weighting schemes are introduced. However, in most cases, it can be seen that those types of weighting schemes basically employ more weight to one particular feature and those cannot be appropriate in the presence of conflicting attributes specifically for these types of constrained crowdsourcing-based applications. Moreover, finding the appropriate weighting schemes as well as finding the normalization criteria are two complex tasks. Besides it, limited research is concerned about the design of a proper positive ideal solution [22], especially for the constrained crowd opinions, when the available option sets are undefined. In the traditional TOPSIS models, the positive ideal solution is considered as the maximum value that becomes 1 after min-max normalization for the benefit criteria (i.e., 1 that should be maximized). Similarly, for the cost criteria, the value for the positive ideal solution is considered as the minimum value (i.e., 0 that should be minimized). But in real-life, specially for conflicting objectives (i.e., benefit and cost criteria), obtaining the two values simultaneously as 1 and 0, respectively, is not realistic. Hence, depending upon these values, the obtained ranking can be erroneous. So, there is a need of making a trade-off between these two values. This similar kind of limitation was identified in the previous work [22]; however, in the proposed approach, class information as a preference order is taken into consideration for the training phase. However, in a crowdsourcing based problem as in our case, obtaining this type of preference information beforehand from the decision makers is not possible.

### Research Objectives

The main research objectives of this current paper are summarized below.

- In this current manuscript, an effort is made to find a proper ranking from the constrained crowd opinions where the option sets are not defined.
- In this work, there is no perfect specification about the weight for the two criteria. For example, in the traditional TOPSIS models, some weights are available from the decision makers. But in this current constrained judgment analysis with an example of urban planning (i.e., locating ATM counters), no weight has been specified by the decision maker (only the priority can be realized and this signifies that a coverage is more important than a deviation), so we cannot directly apply the classical TOPSIS model for group decision making and ranking group members.
- In the general TOPSIS model, the ideal solution can be confusing mainly because of the conflicting nature of objective criteria. Hence, our objective is to find a reference solution that can better represent the near optimal solution that optimizes the two conflicting objectives.
- Apart from the positive ideal solution issues, the different normalization criteria provide diverse results and hence, instead of relying on a single ranking, we are generating multiple rankings and aggregating them to achieve a good ranking.
- In this work, we do not apply any particular weighting scheme, such as entropy weighting or standard deviation-based weighting. On the other hand, it is confusing which metric among many metrics like entropy and a standard deviation should be employed for the proper ranking.
- In addition to comparing the method with standard TOPSIS models with different normalization schemes, we also extensively study the performance of the method with two recently proposed weighted TOPSIS approaches [20,21], which dominate the existing state-of-the-art approaches. The experimental results demonstrate the requirement of multi-objective optimization for crowd judgment problems and the superiority of the proposed approach over the state-of-the-art models is established over the two constrained crowd judgment analysis datasets.

Thus, this work contributes in two folds. First, it finds a better solution from the original constrained crowd responses, in order to optimize the two different conflicting criteria simultaneously. To reward the crowd for motivating the next phase of work in a constrained judgment setting, no study was proposed in the existing literature. In this method, after obtaining the improved solution, a proper positive ideal solution is chosen to employ it in the TOPSIS model. The negative ideal solution is kept the same as the optimal solution i.e., 0 for the benefit criteria and 1 for the cost criteria in order to prevent securing the better ranking of too many outlier solutions. For example, the optimal solution like [benefit attribute cost attribute] = [1 0], may not exist simultaneously all the time in real-life scenarios. The motivation is to use the reference solution in order to employ a positive ideal solution in TOPSIS in a better way for ranking crowd workers. Recently, one work [23] solves the problem of the constrained crowd judgment problem using a differential evolution method, however, no effort has been made to rank the crowd by proper designing of the ideal solution. Hence, the motivations and the proposed model are different from this previous work. In our proposed work, it is not possible to find the accuracies of the crowd workers based on the defined option sets and ground truth solutions as well. Thus, this work aims in finding a good number of solutions from constrained opinions as well as making a proper ideal solution for conflicting criteria to derive a good ranking of crowd.

The rest of the paper is depicted as follows. In Section 2, we represent the state-of-the-art research dealing with this type of crowd judgment problem. In Section 3, the problem formulation is illustrated. Section 4 depicts the proposed method to solve the problem explained thereafter. Section 5 is devoted to experimental design and analysis. Finally, Section 6 concludes the paper including some future directions.

## 2. Related Works

Over the years, a line of research has been carried out to find out the aggregated opinions from multiple crowd opinions to solve different real-life problems [16,24,25,26]. Crowdsourcing as a new aspect of digital employment has the power to alter the nature of the organization as well as yield strategic values for the workers and job providers [2,27,28]. Even though there are several benefits, there is also a possibility of obtaining noisy opinions from them owing to the existence of many non-experts. Among the different models evolved over time, the probabilistic graphical model-based approaches are found to be efficient at simultaneously estimating the crowd workers’ accuracy and an actual answer of the question. One of the approaches produced by Raykar et al. [14] demonstrates that the use of a Bayesian inference algorithm can be efficient at obtaining noise-free judgment. Nevertheless, there are many limitations due to the existence of the local optimality of the solutions. Recently, another work has focused on employing crowds for quality estimation from unstructured texts instead of traditional text documents [29].

In recent years, other research has been proposed to tackle the issue of designing proper strategies for motivating the crowd workers to perform their tasks accurately [30]. Due to the scarcity of the detailed incentive schemes, it was difficult to determine the best rewarding function depending on the quality of the crowd workers. This paper deals with that issue and proposes an aggregation function to validate the crowd workers based on their previous task records. However, in this problem, the overall system is not framed in a constrained judgment analysis setting. In this current constrained judgment analysis model, the design setting is different and there is no provision of collecting repeated opinions from the same crowd workers to provide the answer promptly to the decision makers.

There is recent research that finds qualitative and quantitative results mainly for the document relevance checking tasks [31]. In this work also, a 4-level relevance score is employed and these are: (i) not relevant, (ii) partially relevant, (iii) relevant, and (iv) highly relevant to judging the relevance of the document. Therefore, the questions presented here do not consider the constraint opinion of the crowd workers. From the perspective of the TOPSIS model, the similar kinds of problems related to discovering the best combination of conflicting criteria were investigated in another work [22]. As discussed earlier, the preference-based learning on some class information is performed here to define the positive ideal solution in a better way. In this work, some training process needs to be performed on the basis of some class information. Thus, it trains the model to obtain the pairwise preference depending upon the class information. However, in the new proposed model, there is no provision to have a class label. Another work introduces the TOPSIS model in a different way by using probabilistic linguistic terms [32] and the application is performed in supplier selection problem of new agricultural machinery product. However, introducing this type of linguistic terms is not possible in this constrained crowd opinions. Recently, two modified weighted TOPSIS methods were proposed in the domain of wireless sensor networks [20]. Here, two modified strategies based on entropy (abbreviated as PE-TOPSIS) and a standard deviation (abbreviated as PSD-TOPSIS) are proposed to minimize the nonessential handover and radio link faults in the 5G heterogeneous network. This PE-TOPSIS method introduces a new relative closeness function to find the distance with the ideal solution. It demonstrates the improvement over the other existing models to increase the throughput of the systems minimizing the number of radio link failures. It is seen that PE-TOPSIS generally applies more weight on a particular feature that cannot be fair in the presence of conflicting attributes concerning crowd judgment. More importantly, finding the appropriate weight over the different conflicting objective functions for this type of constrained crowd opinions is a challenge. Another recent work employs entropy in a different way to find the appropriate weight over multiple attributes and demonstrates it efficiency over other existing approaches in the domain of lean construction [21]. The motivation of the work is to find the important drivers based on three sustainability criteria, namely, economic, social, and environmental for successful lean construction. However, this type of application of modified TOPSIS methods have not been explored in the field of constrained crowd judgment analysis scenario in the literature. Alongside, from the perspective of crowd judgment analysis, the work mentioned in [11] introduces the new problem of judgment analysis, while there was no defined option set. However, in this solution approach, the Bayesian binning [33] is used to define the option set and it raises a difficulty as some similar opinions (not exact opinions) are merged, while producing the bins, and this may cause some loss of information. Besides this, the motivation here is not to rank the crowd workers to motivate themselves for better annotations. Another recent work [34] employs the Markov chain-based method to find the ranking for crowds; however, in this work also, the voting method is applied which is not feasible for the constrained opinions of crowds. In our work, we first try to generate the improved solutions optimizing the two conflicting objectives from the constrained crowd responses by proposing a multi-objective optimization algorithm and then find a technique to determine the appropriate ideal solution to enable the TOPSIS model for ranking the crowd in a better way. On the other hand, we do not rely on a particular weighting scheme, rather, along with the proposed ranking we find multiple rankings from traditional TOPSIS models with two normalization schemes and then the final ranking is obtained by aggregating all the ranking. The efficacy of the proposed model is demonstrated with the two real-world datasets for urban planning.

## 3. Problem Formulation

Inspired by the work on ’constrained judgment analysis’ [11], we consider a set of questions $Q=\{q_{1},q_{2},\ldots ,q_{t}\}$ and a set of annotators $A=\{a_{1},a_{2},\ldots ,a_{n}\}$. Here, both the terms “crowd workers” and “annotators” are used interchangeably. The set of opinion vectors is $O=\{\left\{\left(o_{1j}^{11},o_{1j}^{12},\ldots ,o_{1j}^{1m}\right),\left(o_{1j}^{21},o_{1j}^{22},\ldots ,o_{1j}^{2m}\right),\ldots ,\left(o_{1j}^{k1},o_{1j}^{k2},\ldots ,o_{1j}^{km}\right)\right\},\left\{\left(o_{2j}^{11},o_{2j}^{12},\ldots ,o_{2j}^{1m}\right),\left(o_{2j}^{21},o_{2j}^{22},\ldots ,o_{2j}^{2m}\right),\ldots ,\left(o_{2j}^{k1},o_{2j}^{k2},\ldots ,o_{2j}^{km}\right)\right\},\ldots ,\left\{\left(o_{nj}^{11},o_{nj}^{12},\ldots ,o_{nj}^{1m}\right),\left(o_{nj}^{21},o_{nj}^{22},\ldots ,o_{nj}^{2m}\right),\ldots ,\left(o_{nj}^{k1},o_{nj}^{k2},\ldots ,o_{nj}^{km}\right)\right\}\}$, for any particular question *j*, where $o_{nj}^{km}$ denotes the opinion provided by the *n*th annotator for the *k*th dimension of the *m*th component of the question. Between any pair of components, a relation is needed to be maintained and it is considered as a constraint.

Thus, the objective of the problem is two fold in our work. Firstly, from these constrained crowd opinions, the improved constrained satisfying solutions representing a better trade-off between conflicting objectives are derived. Secondly, the objective is to find a realistic positive ideal solution that enables us to obtain the better ranking among the crowd workers. A simple example of the annotation procedure and problem formulation is provided below.

**Figure 1:**
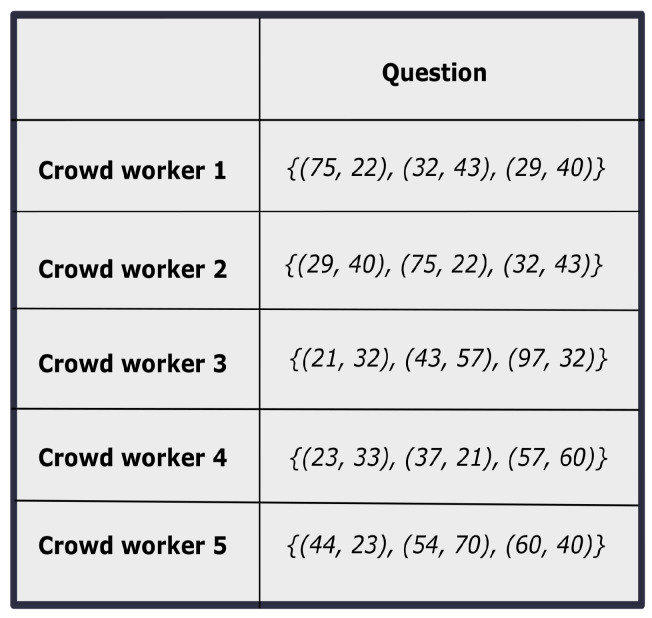
The sample response matrix for the constrained crowd opinions.

An annotation procedure can be considered as a 4-tuple (Q,A,O,τ) that consists of (i) a set of questions *Q*, (ii) a set of annotators *A*, (iii) a set of opinions *O*, and (iv) a mapping function τ:(Q×A)→O. The objective is to obtain the final aggregated judgment of all the questions in *Q*. Note that, in the aforementioned problem, there is no predefined option set, instead of that, only ranges of the options are available. A sample response matrix with different crowd opinions is shown in Figure 1. It can be seen that there are three components having two coordinate values for each component of a sample question. Thus, the objective of the problem is in two folds in our work. First, the improved constrained satisfying solutions that can make a better trade-off between conflicting objectives are observed. Then, based on that improved solution that optimizes both the objective functions, the proper ideal solution of the TOPSIS model [35] is developed. Finally, the crowd workers are ranked based on the ideal solution. Therefore, on the one hand, the objective is to find a better solution from the original crowd solution. On the other hand, the objective is to find a realistic positive ideal solution that enables us to obtain a better ranking among the crowd workers.

## 4. Proposed Model

**Figure 2:**
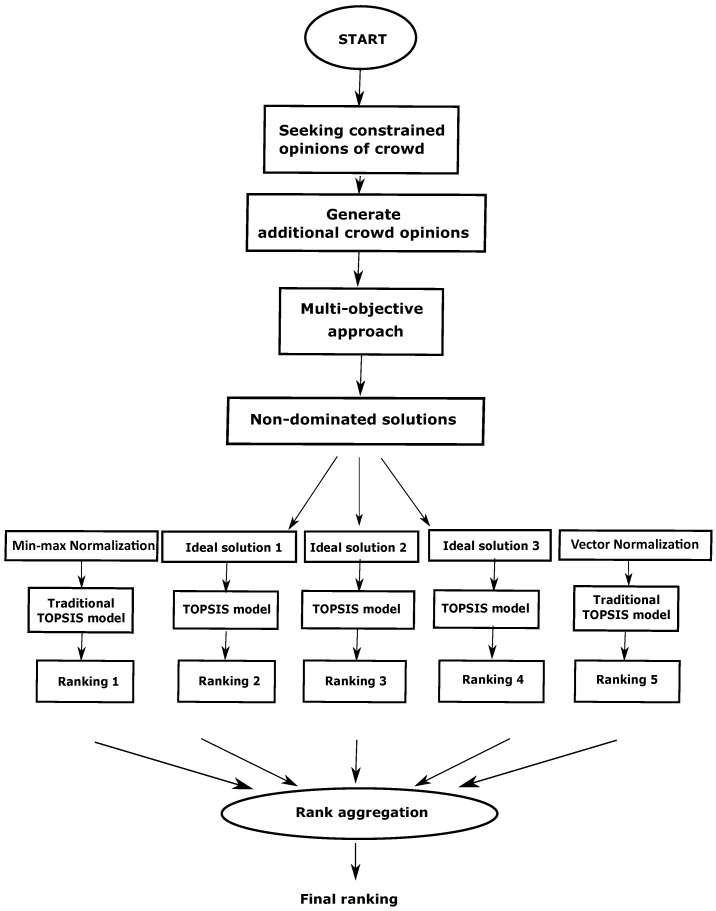
The overall flowchart of the proposed approach for ranking the crowd workers.

In this section, the proposed methodology is discussed in different subsections. As mentioned in the earlier section, the proposed method to find the final ranking from the crowd opinions consists of three main stages. First, as there is no correspondence between the labeling of the crowd worker, therefore the relabeling of the responses are performed (Section 4.1). Then a multi-objective evolutionary-based approach is presented to find a better reference solution (Section 4.2) comprising two objectives. In order to brief the traditional TOPSIS model, we describe it in Section 4.3. The reference solution obtained in Section 4.2 is used as the positive ideal solution of the TOPSIS Model (Section 4.4). Then multiple rankings are generated based on the traditional TOPSIS models (with different normalizations) as well as modified TOPSIS Model. Finally, a rank aggregation method is incorporated to find the final ranking (detailed in Section 4.4). The subsequent steps of the proposed method for the aforementioned problem are detailed hereafter. The pictorial representation of the overall workflow is demonstrated in Figure 2.

### 4.1. Relabeling of Inconsistent Crowd Solutions

In this above-mentioned constrained crowd judgment analysis problem, the main challenge is that there is no correspondence between the different crowd responses. To illustrate, in this current problem, the opinions about the 2D coordinate values of *k* locations are solicited from the crowd workers. Here, each *k* location can be treated as *k* components. Hence, each opinion of the crowd worker represents the *k* 2D coordinate values. But as there is no defined ordering between the *k* components, so one crowd worker’s opinion for the first component can be the other worker’s opinions for the second component. For example as shown in Figure 1, the two solutions provided by Crowd worker 1 and Crowd worker 2 are same, but their representations are different. Here we notice that Crowd worker 1’s third component coordinate value is same with Crowd worker 2’s first component coordinate value. Similarly, the coordinate values are same for the first component of Crowd worker 1 and the second component of Crowd worker 2. Therefore, it is always needed to bring the correspondence between the solutions.

In order to bring forth the correspondence between the coordinate values of the crowd workers’ solutions, first, one representative solution from all the crowd solutions are chosen. A sample question can be like “An organization expresses their interest to open three ATM counters in a locality and for that purpose what should be appropriate locations?”. Thus, there are two objectives (i.e., the coverage enclosed by the three location points as the first objective and the deviation of the solutions from the mean as the second objective), which are specified by the decision makers, but not revealed to the crowd workers. The reference solution is selected based on the first objective function value, as it has the higher priority. After choosing the reference solution, all the crowd solutions are transformed based on the labeling of the reference solution. In order to make the correspondence with respect to the reference solution, first, the Euclidean distance from the first component of the reference solution is computed with the each of the components of the other crowd workers’ solutions. That component having a minimum distance with the first component of the reference solution is finally selected as the first component of that crowd worker’s solution. Then, that component is not considered in the next step. In the second step, again as the similar way, the distance is computed from the second component of the reference solution with the rest of the other components of the crowd solution and finally the component for which the distance is minimum is selected as the second component of that crowd worker’s solution. In that way, all the components of the particular crowd worker’s solution is relabeled based on the reference solution and finally all the crowd workers’ solutions are relabeled accordingly.

### 4.2. Proposed Multi-Objective Formulation

This section illustrates the proposed method utilizing the NSGA-II [36] framework for generating a set of near Pareto-optimal solutions and it is described in a step-by-step manner.

#### 4.2.1. Encoding Scheme

**Figure 3:**
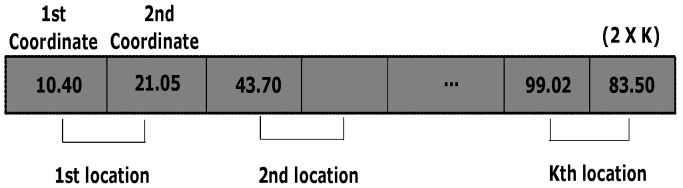
The scheme of encoding a chromosome.

Considering the crowd workers is needed to select *k* locations based on their perceptions. Hence, the solution comprises 2D coordinate values of *k* locations. Each cell value of the chromosome expresses a specific coordinate value (e.g., either *X* or *Y* coordinate of a 2D case) for a specific location. Therefore, in case of *k* locations for the 2D coordinate, the length of a chromosome should be 2k with real values. In this problem, a grid map of a particular region is shown to the crowd workers where the $\left\{X_{min},X_{max},Y_{min},Y_{max}\right\}$ are shown to them. Therefore, when the initial population is created randomly, these margins are taken into consideration to restrict the generation of infeasible solutions falling outside of the margin. Thus, the bounding box is employed with the specified margins in order to keep the random solutions in that specified zone for all the components. The encoding scheme of chromosome is shown in Figure 3.

#### 4.2.2. Initial Population

The initial population is generated with the whole set of the original crowd solutions. Additionally, we generated some other solutions randomly guided by the crowd workers to explore the search space more and remove any bias towards a particular original crowd solution. On the other hand, after creating the initial population, we need to relabel the crowd workers’ opinions as described in the Section 4.1.

#### 4.2.3. Selection

In this step, the chromosomes are selected for further breeding, guided by the perception of the crowd workers. In general, a binary tournament selection method is adopted, however, in a multi-objective scenario, the crowded binary tournament selection strategy is employed in expectation of maintaining diversity in the population.

#### 4.2.4. Crossover

Crossover is a genetic operation that alters the genetic code among the two individual chromosomes. In this process, a small probability $p_{c}$ within the range [0–1] is employed and a multi-point crossover is performed using a binary mask.

#### 4.2.5. Mutation

Every chromosome runs through a mutation process according to a little mutation probability $p_{m}$. In this context, a random value ranging between [0–1] is added or subtracted into one or multiple cells of the chromosome.

#### 4.2.6. Elitism

In order to keep the best chromosome of every generation in the population, the elitism method is automatically incorporated in the NSGA-II method by combining the parent population with the child population. Additionally, we include the best solution evolved in the current generation to preserve in the next generation. The best solution is chosen depending on the ratio of the first objective to that of the second objective function. Thereafter, the constraint satisfying condition is checked and finally, the maximum value is chosen as the best chromosome to be preserved for the next generation.

#### 4.2.7. Choice of Objectives

In this current work, the two conflicting objective functions are considered. The first one is the coverage enclosed by the facilities located at *k* points in the city. The proper planning represents that all the facilities should be well distributed across the region so that maximum people can get benefit out of it. Hence, the coverage plays an important role in this context. On the other hand, to remove any kind of bias towards a particular solution, the deviation of the solution from the mean is calculated and this objective function is considered as the second objective function. In this context, the objective is to maximize the first objective function (i.e., the coverage) and to minimize the second objective function (i.e., the deviation from the mean). Here, the mean solution is computed component-wise and these values are subtracted from the crowd solutions. The lesser deviation means the quality of the solution is good and it is close to the mean solution, thus it should be minimized.

#### 4.2.8. Repairing Solutions

During each generation, while traversing through the different genetic operations specially for crossover and mutation, the genetic information of the chromosomes is changed. Therefore, the cell values of the chromosomes are altered. However, according to the crowd opinions, the solutions should be bounded by some coordinate values which keep the solutions in some feasible regions. Therefore, to avoid generating the infeasible solutions, the altered values of chromosomes are repaired based on the original crowd responses. However, before repairing, the original crowd solutions are relabeled (as described in Section 4.1) to bring the correspondence among them. After that, the scaling procedure is applied and it is explained below.

Let $C=\{c_{1},c_{2},\ldots ,c_{m}\}$ denote the coordinate values (either X or Y coordinate), after obtaining the opinions from the original crowd at the very beginning phase of the proposed model. Suppose C is the vector of *m* crowd opinions for any one of the coordinate of one ATM counter. Now let $S=\{s_{1},s_{2},\ldots ,s_{m}\}$ be the coordinate values of the chromosomes for a particular coordinate of one ATM counter, while passing through the different genetic operations. Now, these values of S are needed to be scaled based on the original crowd solutions C.

Hence, the formula to adjust the value $s_{i}$ of S to a new value $s_{i}^{\prime }$ is written below.

(1) $$s_{i}^{\prime }=\min\left\{c_{1},c_{2},\ldots ,c_{m}\right\}+\frac{s_{i}-\min\left\{s_{1},s_{2},\ldots ,s_{m}\right\}}{\max\{s_{1},s_{2},\ldots ,s_{m}\}}*w,$$

where, $w=\max\left\{s_{1},s_{2},\ldots ,s_{m}\right\}-\min\left\{s_{1},s_{2},\ldots ,s_{m}\right\}$.

In this way, all the other coordinate values of a particular location are scaled. At the last stage, the scaled solutions are merged with the parent population utilizing the idea of non-dominated Pareto front and crowding distance. The algorithm is terminated after a certain number of iterations, while a particular objective function is converged.

### 4.3. The Classical TOPSIS Model

TOPSIS [35] produces a ranking for the alternatives depending upon a set of decision criteria. The ranking is performed by choosing the alternative that simultaneously has the shortest distance from the positive ideal solution (PIS) and the longest distance from the negative ideal solution (NIS). The positive ideal solution is that maximizes the value for the benefit criteria, i.e., more means better, whereas, the negative ideal solution is that minimizes the value of the cost criteria, e.g., lower means better.

The different steps for the TOPSIS model are described in below.

- Construct the decision matrix
  Suppose, $X={\left(x_{ij}\right)}_{m\times n}$ be a decision matrix, where *m* be the number of alternatives, *n* be the number of criteria, and $x_{ij}\in R$. The criteria of the function can be of conflicting types, that is, benefit functions (higher is better) or cost functions (lower is better).
- Calculate the normalized decision matrix
  This step converts the dimensional attributes to non-dimensional attributes that make provisions for comparisons between the criteria. The reason is that various criteria are traditionally measured in different units, hence the scores of the criteria should be measured in a normalized scale. The normalization can be of various types and two of them (i.e., vector normalization and min-max normalization) are discussed here. The normalized value $n_{ij}$ and $m_{ij}$ using these two methods can be calculated as Equations (2) and (3).
  (2) $$n_{ij}=\frac{x_{ij}}{\sqrt{\sum_{i=1}^{m}x_{ij}^{2}}}$$
  (3) $$m_{ij}=\frac{x_{ij}-\min_{i}x_{ij}}{\max_{i}x_{ij}-\min_{i}x_{ij}}.$$
- Calculate the weighted normalized decision matrix considering *w* is a weight vector
  (4) $$v_{ij}=n_{ij}\cdot w_{j},i=1,2,\ldots ,m\;and\;j=1,2,\ldots ,m\;\mathrm{so}\;\mathrm{that},\sum w_{i}=1.$$
- Calculate the positive ideal solution and the negative ideal solution
  The positive ideal solution ($A^{+}$) is of the form:
  (5) $$A^{+}=\left(v_{1}^{+},v_{2}^{+},\ldots ,v_{n}^{+}\right)=\left(\max_{i}v_{ij}|j\in I\right),\left(\min_{i}v_{ij}|j\in J\right).$$
  The negative ideal solution ($A^{-}$) is of the form:
  (6) $$A^{-}=\left(v_{1}^{-},v_{2}^{-},\ldots ,v_{n}^{-}\right)=\left(\min_{i}v_{ij}|j\in I\right),\left(\max_{i}v_{ij}|j\in J\right).$$
  where *I* is associated with the benefit criteria and *J* with the cost criteria, *I* = {1, 2, …, *m*} and J={1,2,…,m}.
- Compute the separation measure $\left(d_{i}^{+}\right)$ and $\left(d_{i}^{-}\right)$ with respect to the positive ideal solution and the negative ideal solution.
  The separation of each alternative from the positive ideal solution and the negative ideal solution is computed by:
  (7) $$d_{i}^{+}={\left(\sum_{j=1}^{n}{\left(v_{ij}-v_{j}^{+}\right)}^{p}\right)}^{\frac{1}{p}},i=1,2,\ldots ,m$$
  (8) $$d_{i}^{-}={\left(\sum_{j=1}^{n}{\left(v_{ij}-v_{j}^{-}\right)}^{p}\right)}^{\frac{1}{p}},i=1,2,\ldots ,m.$$
  Here, p≥1 and if p=2, then it becomes a traditional Euclidean metric.
- Calculate the relative closeness with respect to the positive ideal solution
  The relative proximity $R_{i}$ of the *i*th alternative $A_{j}$, with respect to $A^{+}$ is derived by
  (9) $$R_{i}=\frac{d_{i}^{-}}{d_{i}^{-}+d_{i}^{+}},\;\;1<i<m.$$
- Rank the preference order or select the alternative closest to 1
  In this way, the rankings of all the alternatives are performed.

### 4.4. Modified Ideal Solution for TOPSIS

In the traditional TOPSIS model, suppose the positive ideal solution for the two attributes is considered as [1 0], considering the first attribute as the benefit criteria and the second attribute as the cost criteria. Note that, while performing normalization, the cost attribute is transformed to the benefit attribute, thus [1 0] will be written as [1 1] from now on. We obtain the improved solutions, after applying the proposed multi-objective optimization algorithm. Thereafter, we choose one solution based on the original crowd opinions to filter the generated solutions with an aim to identify the proper ideal solution. This reference solution (chosen from the original crowd) is selected with respect to one objective function, which has the highest priority. Now, based on the reference solution, all the non-dominated solutions irrespective of all the ranks are sorted based on the greater or equal value with respect to the first objective of the reference solution and lesser or equal value with respect to the second objective of the reference solution. Thus, all the solutions having higher value based on the first objective and lesser value based on the second objective are filtered. After that, the average values for both the objectives of all the filtered solutions are computed. Thereafter, to obtain the normalized value, this average solution is put together with the original crowd solution and min-max normalization is performed. Finally, this normalized value of that solution is considered as the ideal solution. In this way, the positive ideal solution can be generated in the form of [1 *x*], where 0≤x≤1. Here, the first item is for the benefit criteria and the second item is for the cost criteria. After obtaining the ranking utilizing the ideal solutions, we perform the aggregation based on Markov chain-based MC4 [37,38]. In this method, we find the ranking of crowd workers by executing the proposed method three times. Then we also performed ranking by the traditional TOPSIS model using two normalization procedures. Finally, we apply the rank Markov chain-based aggregation method.

## 5. Experimental Design and Results

In this section, the two crowdsourced datasets for urban planning, which are used in this paper for the experimental purposes, are described. The first dataset is collected from [23] and the second dataset is collected from [11]. The experiments are performed in MATLAB 2013a and the environment is an Intel(R) CPU 2.4 GHz machine with 8 GB RAM running Windows 10.

### 5.1. Dataset Preparation

To simulate the experiments, we prepared the two different datasets for urban planning by obtaining the responses from crowd. The first dataset was prepared by posting a grid map of Ulsan National Institute of Science and Technology (UNIST) campus [23]. In this grid map, there is a combination of diverse geographical features like forest, sports ground, and lake. Then, a question is posted by showing the grid map to obtain the response from crowd. In the online forum, the posted question is like that “An organization wishes to install three ATM counters inside the campus of UNIST and so what will be the possible location to install the three ATM counters?”. The additional instruction in this question is that there will be at least 20 units distance between any two ATM counters. Therefore, the response here is an opinion of triplet, where each response signifies the three possible locations of ATM counters. Meanwhile, as responding the question, it is also required to satisfy the additional instruction i.e., the constraint. In this way, 24 crowd workers registered in the online portal. Among them, three workers provided their responses only for one coordinate and one crowd worker provided no response. Finally, out of 20 responses, two crowd workers’ responses are constrained violating solutions.

On the other hand, to prepare the second dataset, we use another grid map of a state of India mentioned in [11] posting the question like “A top-ranked US university is willing to introduce three extension centres at that state and what will be the three possible locations for this?”. Here also, the constraint is maintaining a specific distance like 30 units between any two extension centres. Therefore, while providing the responses, crowd workers are also needed to satisfy the constraint. In this regard, many crowd workers may spam the process without knowing the exact answer and providing the responses without maintaining the constraint. Moreover, some crowd workers may provide their responses nearest to their home town instead of considering the global aspects. In this dataset, there are 20 crowd workers responding to the question and among them 18 satisfied the constraints.

### 5.2. Study on the First Dataset

In order to conduct the experiment to derive the near optimal solutions from the original crowd optimizing both the criteria, for the first dataset, we generate a total of 80 solutions obtained from crowd. As in this dataset, 20 original crowd opinions are finally considered, therefore, from these 20 solutions, another 60 random solutions are evolved. In this process, the maximum and minimum values of each coordinate of all the crowd solutions are identified. These values are used to refine the random solutions within these ranges and all the solutions are relabeled based on a reference solution of original crowd (as described in Section 4.1). The reference solution of original crowd is chosen based on the first objective. After that, all the solutions are processed based on the two objectives using the proposed multi-objective approach. Finally, the solutions are selected based on the condition of satisfaction of constraint.

In order to evaluate the performance of the model, initially, the goodness of all the crowd workers’ solutions are evaluated. For this purpose, we computed the values of two objective functions, when each one is compared to all the other solutions. The experimental results, when each crowd solution is compared with all the other crowd solutions, are reported in Table 1. Here, only the top-10 solutions according to the first objective are reported. It can be noticed from the table that Solutions 1, 2, and 3 have a better compromise between the two objectives. It can seen that Solution 1 has the highest value in terms of Objective 1 and this solution can be treated as the best solution. We cannot treat Solution 10 as the best, because although it has lower value in terms Objective 2, but Objective 1 value is very less. Similarly, Solutions 8 and 9 can also not be considered as the good solution. However, obtaining the proper ranking is difficult according to the existing model based on TOPSIS due to the presence of conflicting objectives.

We present the solutions for the first dataset obtained after applying the algorithm after 50 generations having 80 population and the top-10 solutions are reported in Table 2. It can be observed from the table that many improved solutions in terms of both the objectives have been achieved. First of all, it can be noticed that there are many solutions that have higher values than the best solutions, that is, Objective 1 of Table 1. From Table 2, we can also view that there are some solutions i.e., Solutions 3, 4, 5, 6, 8, and 9 have better values in terms of both the objectives than Solutions 1, 2 and 3 of Table 1. Thus, it can be easily realized that many improved solutions are generated by using the proposed method. To perform extensive analysis, we conduct another set of the experiments for the other set of parameters, that is, number of generations = 60 and population size = 100 and the results are reported in Table 3.

The performance is also analyzed with population size 100, number of generation 60, and the top-10 solutions (as shown in Table 3). For this scenario also, it can be noticed that all the crowd solutions have greater values in terms of the first objective than Solutions 1, 2 and 3 of Table 1. It is seen that the first objective value is higher than all the three best solutions of Table 1. There are also many solutions, which have higher values in respect of both the objectives for the same generations and population size and these corresponding experimental results are reported in Table 4.

After obtaining the solutions from the proposed multi-objective approach, in the subsequent phases, with an aim of obtaining a positive ideal solution, the solutions are filtered based on a reference solution selected from the original crowd. The reference solution from the original crowd is selected based on the higher value in respective of the first objective. For example, Solution 1 of Table 1 is considered as the reference solution for the first dataset. Thereafter, the procedure described in Section 4.4 is applied to filter only those solutions, which have higher than or equal value to the first objective and lower than or equal value to the second objective of the reference solution. Hereafter, the constraint satisfying solutions are chosen.

#### Further Illustration on Choosing Modified Ideal Solution

To illustrate the last phase of the algorithm as described in Section 4.4, a sample example is provided here. It can be observed that in Table 1, there are 10 crowd solutions and as the priority is given on Coverage so according to the first objective, the Solution 1 having highest value in the first objective is considered as the reference solution. Now, after applying the proposed algorithm, it can be noticed that there are many solutions having better values than the reference solution obtained from the original crowd solutions. So in Table 3, among many improved solutions obtained by the proposed approach, 10 sample solutions having greater value than the reference solution (as the value of the first objective is higher than 1.2, that is, first objective of Solution 1) are demonstrated. Now from these solutions obtained from the proposed approach, as mentioned in Section 4.4, some solutions are filtered based on the reference solution. For example, we can see that the reference solution of original crowd Solution 1 of Table 1 has first objective value as 1.2 and second objective value as 0.0499. After applying the algorithm, although we obtained many solutions having better values in both the objectives (optimized simultaneously), now we filter the solutions having higher value than the first objective i.e., 1.2 and lower value than the second objective (i.e., 0.0499) of that reference crowd solution. Hence, we can notice some of the solutions after filtering based on this criteria are removed from filtered solutions. The filtered solutions having higher or equal value than 1.2 and lower or equal value than 0.0499 (based on the reference crowd solution) are demonstrated in Table 4. Now to obtain the proposed ideal solution compromising the two objectives, the average values of all the filtered solutions (as a sample example shown in Table 4) for these two objectives are considered.

Finally, this average value is considered with all the crowd solutions and normalization is done to find the proposed Ideal solution within the range [1–0] for both the objectives. We can visualize easily that in reality, if the crowd solution has higher value in terms of coverage (first objective) then automatically the deviation from mean is also increased. For example, we cannot treat Solution 13 of Table 5 as good solution although it has lowest value (i.e., 0.0157) in terms of second objective, as the first objective becomes very less i.e., 0. Hence, a proper estimate of the solution optimizing both the objectives is required to derive the proposed Ideal solution. So this solution cannot be considered as the ideal solution. On the other hand, the reference crowd solution having first objective as 1.2 and second objective as 0.0499 cannot be considered as the proposed ideal solution, because there can be existence of some solutions having first objective greater value than 1.2 and less value than 0.0499. Therefore, the advantage of choosing this solution in this way clearly demonstrates that it balances the two objectives and finally produces the proper ranking. Moreover, if we simply consider the best solution 1.2 for first objective (considering solution 1 of Table 1) and second objective as 0.0157 (Solution 13 of Table 5) that cannot be realistic. The reason is that if the coverage becomes high the deviation may not necessarily be near to zero most of the time according to crowd solutions. Therefore it exhibits the advantages of considering the proposed solution in this above mentioned way. The experimental results reported in various Table 5, Table 6, Table 7, Table 8, Table 9, Table 10, Table 11 and Table 12 considering different Ideal solutions along with the proposed Ideal solution (treating both the objectives simultaneously after Multi-objective optimization approach) also clearly demonstrates the effectiveness of this approach. Moreover, as discussed in the traditional TOPSIS model, the ideal solution simply considers highest value for the first objective and minimum value for the second objective without considering the optimized value simultaneously, But in the real scenario, especially for crowdsourcing-based policy making, it is highly required to obtain the solution that optimizes the multiple objectives concurrently.

**Figure 4:**
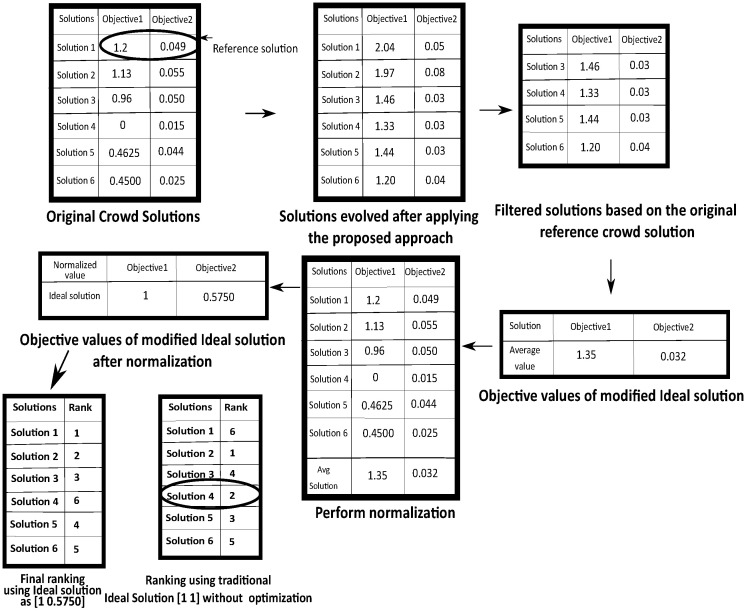
The overall flowchart for identifying the modified Ideal solution.

The overall flowchart of the whole process over a sample example is depicted in Figure 4. Here it can be noticed that in the 3rd step, some of the solutions are filtered which have better or equal value than the original reference crowd solution in terms of first objective (i.e., solution 1 of first table in Figure 4) and lower or equal value than the same solution in the second objective. Finally the average value is taken for both the objectives and it can be treated as the modified Ideal solution. It can be seen that this modified Ideal solution has better value in terms of both the objective values then the reference crowd solution compromises both the objective functions. Finally, this solution is employed for normalization and performing proper ranking.It can be noticed that computing the Ideal solution in this way produces the ideal solution as [1 0.5750] for the two objectives and ranking of those solutions becomes {1,2,3,6,4,5} for these 6 solutions. However, if this is not considered and only the traditional Ideal solution is chosen as [1 1] then the ranking of the 6 solutions becomes {6,1,4,2,3,5}. So it can be seen that Solution 4 has the worst value in terms of first objective (having higher priority) but it received second rank as the second objective value of this solution is very less. However, as mentioned before, this solution 4 cannot be considered as a promising solution. Although, the ranking obtained from the modified Ideal solution produces the proper ranking keeping the Solution 4 as in 6th Position. Thus the effectiveness and advantages of the method can be observed.

### 5.3. Further Study on the First Dataset

In this dataset, the reference solution selected from original crowd (i.e., Solution 1 of Table 1) has the first objective value 1.2000 and the second objective value 0.0499. The filtered solutions based on the reference solution are reported in Table 4. It can be easily noticed from Table 4 that there are 11 solutions that have better values in terms of both objectives of that reference solution. Note that, from the original crowd solutions, if the first objective becomes the highest, then it is less likely that the second objective becomes the lowest. Therefore, the positive ideal solution cannot be [1 1] for conflicting cases (i.e., for benefit and cost attributes). In this regard as mentioned earlier, while executing the normalization, the cost attribute is transformed to the benefit attribute, thus the classical ideal solution [1 0] has been written as [1 1] here. However, as observed from these results, many improved solutions than the reference solutions can be obtained from the filtered solutions to achieve a proper positive ideal solution. To derive the objective values for the positive ideal solution, an average of all these solutions is computed for both the objectives and finally these average values are considered for the calculation of the positive ideal solution.

After producing the average value from the final filtered solutions, we then normalize all the crowd solutions based on the new average filtered solution and then that normalized value is treated as the positive ideal solution. In this respect, for the proposed model, min-max normalization is used. As the filtered solutions are changed after different runs of the proposed multi-objective genetic algorithm-based (MOGA) model so as to check the performance, the algorithm is executed thrice and different ideal solutions e.g., [1 0.3558], [1 0.4590], and [1 0.3781] are obtained. Thereafter, based on these ideal solutions, the ranking of the crowd workers can be determined using the TOPSIS model. The experimental results obtained for three executions of the proposed model (abbreviated as MOGA) are reported in Table 5. In this table, the alternatives are the different solutions obtained from the crowd and the criteria are the two objective functions. It can be noticed in different executions of the proposed model that there are minimal changes in the ranking. In order to compare the performance of the proposed ranking, we also apply the traditional TOPSIS models with two types of normalization procedures. Min-max normalization and vector normalization are applied in this step. Note that, in the proposed model, due to the unavailability of the explicit weights over the multiple criteria, no weight has been assigned in traditional TOPSIS.

To further analyze the experimental results mentioned in Table 5, we visualize that Solution 13 has coverage 0, but it is ranked the 3rd by TOPSIS (vector normalization) and the 5th by TOPSIS (min-max normalization). First of all, the positions of different crowd workers in different rankings obtained by traditional TOPSIS are different. So, obtaining a proper decision about the perfect ranking of the crowd workers are difficult, if only TOPSIS models are considered. The reason that it uses [1 0] as the positive ideal solution as the benefit and cost attribute, although obtaining perfect 1 (benefit attribute) and 0 (cost attribute) concurrently in real-life is highly difficult. Note that, while performing normalization, the cost attribute is transformed to the benefit attribute, thus [1 0] has been written as [1 1] here also. Again, this 13th solution cannot be treated as a good solution due to the 0 coverage value. This point can be identified by the proposed ideal solution obtained by the multi-objective approach by positioning this Solution 13 as the 7th rank. Actually, this solution should be ranked near to medium, although it is positioned the third according to TOPSIS vector normalization. On the other hand, the proposed approach does not rely on a single ranking solution, rather we apply an effective rank aggregation algorithm [38,39] to produce a better ranking from multiple rankings. From the aggregated ranking, it can be found that Solution 13 is in Position 7. Additionally, as the similar ranking is obtained from different executions of the proposed ideal solutions, we choose one ranking from it (i.e., the first one), then again the rank aggregation algorithm is performed. From this ranking also, Solution 13 is ranked as the 5th and it is not at the top rank i.e., 3 as obtained by the TOPSIS model (vector normalization).

It can be noticed that Solution 1 has the higher coverage than Solution 13, but it is ranked worse than Solution 13. The reason can be due to the larger deviation. Another point is that, Solution 8 is the 2nd according to TOPSIS (min-max normalization) due to the lower value in respective of the second objective. However, there are many good solutions (according to the first objective) like Solutions 1, 17 and 18, but due to their high value in the second objective, they are getting a worse rank than Solution 8 in case of min-max normalization. Although, this Solution 8 is ranked the 5th, if we change the normalization procedure to vector normalization in the same TOPSIS model. Even if we apply the proposed ideal solution, then we can obtain the same ranking as 5 as found in vector normalization. Therefore, the aggregated ranking removes the anomaly for Solution 8 as it provides the 5th and 4th rankings, respectively.

In addition with comparing the performance with respect to the traditional TOPSIS models with different weighting schemes, we also extensively compare the performance of the proposed method with three most state-of-the-art approaches [20,21]. The experimental results obtained from these three approaches are reported in Table 6. This work [21] (termed as Weighted TOPSIS) introduces the entropy-based weighting scheme for finding better ranking in order to obtain the ideal solution. It is seen from the Table 6, Solution 20 is ranked as 6, although the first objective (i.e., coverage) is medium good but the second objective is also not so good. Therefore, Solution 20 should not be ranked in a good position (i.e., 6) in the ranking. Basically, Solutions 17, 18, and 1 have higher ranks as they have very high values in terms of the first objective, although they have poor second objective function values. Actually, in the weighting scheme, 0.8997 is considered as the weight for the first objective function and 0.1003 is considered as the weight for the second objective function. Now as the maximum weight is kept towards the first objective function, hence the solutions are mainly sorted based on the first objective without considering both the objective functions. Another interesting observation, we notice Solution 5 and Solution 8 have very close values in terms of the first objective values, but the second objective value of Solution 5 is much worst than Solution 8 as nearly double. But in this method [21], owing to applying more weight on the first objective function, Solution 5 is kept as a better position than Solution 8 that cannot be a good compromise for the conflicting objectives. Similarly, Solution 13 has been ranked as 20th as its first objective value is 0. However, its second objective function value is minimum, hence, this solution should be ranked as medium if both the solutions are considered. In this problem, although the first objective function i.e., a coverage value is more important and should be prioritized more, even so, the second objective value cannot be ignored totally. Our proposed work provides the objective values of the different crowd solutions along with the appropriate ranking to the decision makers. From the two objective values of all the crowd solutions and the appropriate ranking compromising both the conflicting objectives by our method, the decision makers can have a choice to select the most appropriate solutions according to their need. Thus, this demonstrates the novelty and applicability of our proposed method. Moreover, a weighting scheme in this way mentioned in the [21] cannot be feasible as most of the weight is imposed on a single function (i.e., first objective) completely neglecting the second objective function. In a similar fashion, the ranking obtained by PE-TOPSIS and PSD-TOPSIS [20] rank the solutions based on the entropy (with the modified distance measure compared to the ideal solution) and a standard deviation feature. In the PE-TOPSIS method, the weight for the first objective is 0.4593 and the weight for the second objective is 0.5407. In the PSD-TOPSIS method, the weight distributions are 0.7188 and 0.2815 for the two objective functions respectively. It can be seen from the Table 6, the PSD-TOPSIS method totally impose much weight on the first objective thus neglecting the second objective value. The ranking obtained by PSD-TOPSIS method demonstrates that Solution 15 is in a better position than Solution 3 as maximum of the weight is applied on the first objective. It can be noticed that Solutions 15 and 3 have closer values in respect of first objective but the second objective value Solution 3 is much better than the Solution 15, thus Solution 3 should be in a better ranking. Interestingly, the other ranking proposed by PE-TOPSIS keeps that Solution 3 in a better position than Solution 15. Thus the weight of the conflicting objective functions in the ideal solution cannot be treated as good as demonstrated by these methods for this constrained crowd-based judgment analysis problem. In fact, the proposed model as illustrated in Table 5 relies on the multiple rankings compromising the multiple objectives and finally derives an aggregated ranking in order to produce a better ranking.

To investigate the performance of the method, another set of the experiments with different population sizes and generation numbers are carried out and the results are reported in Table 7. In this method, instead of choosing the average solution from the filtered solution (as described in the Section 4.4), we choose only one solution that has the minimum value in respective of the second objective value. As described in Section 4.4, the filtered solution is based upon a reference solution that was selected from the original crowd having the highest value in the first objective. Thus, it ensures that the final solution selected for the ideal solution has higher or equal value in terms of the first objective than all the solutions and lower than or equal to the reference solution. After applying this method, it can be observed from Table 7 that the proposed ideal solutions are [1 0.5569], [1 0.5262], and [1 0.5824] for the 50 generations and 100 population. Employing that ideal solutions, the ordering we obtained is similar to the ranking obtained using the average solutions. However, in different executions of the method, it produces different ordering in majority cases, hence it is less stable than the previous method. Although as the result obtained from this method, we notice that the 13th solution is still in the 7th position and not like in the very early position as obtained by TOPSIS vector normalization method. Similarly, applying the proposed ideal solution, Solution 8 is placed in the 5th position that is also interesting. The reason is that Solution 8 is placed in the 2nd position according to TOPSIS min-max normalization, hence the effectiveness of the proposed method can be better realized.

### 5.4. Study on the Second Dataset

In order to perform the experimental analysis for the second dataset, we apply the same algorithm with 80 populations and 50 number of generations. First, the goodness of all the original crowd solutions are examined and the top-10 solutions according to the first objective are mentioned in the Table 8. In this respect, each crowd worker’s solution is compared with all the other crowd workers’ solutions. Experimental results demonstrate that Solutions 1, 2, 3, 4 and 5 can be chosen as the good solutions as they have the higher value in terms of the first objective, although the second objective value is higher in those solutions except Solutions 3 and 4. The solution having very less value for the coverage but very close to the mean solution (i.e., less deviation) cannot be considered as the better solution. Solution 1 has the highest value in terms of the first objective, whereas not so good value in terms of the second objective. First, to test how effective solutions are achieved from the proposed multi-objective approach, we applied the algorithm and the solutions obtained from this proposed approach are reported in Table 9. To make the comparison, we compute the values of two objective functions based on the set of all the original crowd solutions. It can be observed that in Table 8, the original crowd solution has the highest Objective 1 value as 1.3305 and it has Objective 2 value as 0.0258. After applying the algorithm, we obtain multiple improved solutions having the higher value in terms of the first objective than Solution 1 of Table 8 and these values are presented in Table 9. In this table, the top-10 solutions based on the first objective are reported and it can be noticed that Solutions 5, 6, 7, 8, 9, and 10 have better values in terms of both the objectives than Solution 1 (having the highest Objective 1 value) of Table 8. On the other hand, it can also be noticed that Solutions 5 and 7 of Table 9 have the better values in respect of both the objectives than another good solution of original crowd i.e., Solution 2 of Table 8.

For extensive analysis of the proposed model and in order to investigate how improved solutions are generated, we also carried out the experiment with different numbers of generations and populations. Table 10 represents the set of results for population size = 100 and generation number = 60. Here, we represent the top-10 values in terms of Objective 1. From these results also, it can be observed that all of the solutions out of 10, have the higher values in respect of the first objective than Solution 1 of the original crowd as represented in Table 8. Besides it, as the next phase of the model, to find the positive ideal solution, the solutions are filtered out based on a reference solution selected from the original crowd. The reference solution is chosen which has the highest value in terms of Objective 1. Hence, in this dataset, Solution 1 reported in Table 8 is considered as the reference solution. Thereafter, based on the reference solution, we filter the solutions which have higher than and equal value with respect to Objective 1 and lesser than or equal value with respect to Objective 2. Thus, the objective values of the filtered solutions are presented in Table 11. It can be observed that the solutions maintain a well trade-off between the two conflicting objectives. To exemplify, Solution 2 of Table 11 has the highest value in respect of the first objective and it is better than Solution 1 of the original crowd reported in Table 8. On the other hand, Solution 1 of Table 11 has the better value than another good crowd solution i.e., Solution 2 of Table 8, in respective of both the objectives. Moreover, this solution has the higher value for the first objective than Solution 3 of Table 8 and very close value in terms of the second objective. Thus, it demonstrates the effectiveness of the proposed approach. Thereafter, these positive ideal solutions are incorporated in the TOPSIS model to find the ranking of the crowd workers and it is described later.

As the next phase of the model, in order to produce the ranking among different crowd solutions, the proper positive ideal solutions are computed. To perform these, we consider the final filtered solutions and the average value in terms of the first objective and the second objective are computed. For the evaluation purpose, we apply the traditional TOPSIS model with two different types of normalization (i.e., min-max normalization and vector normalization) along with the proposed approach. The proposed multi-objective genetic algorithm (abbreviated as MOGA) is applied thrice and the generated solutions are employed to derive the ideal solutions. According to the traditional TOPSIS model, the positive ideal solutions are [1 1], whereas for the proposed model, the ideal solutions become [1 0.7318], [1 0.7582], and [1 0.7699]. Note that, as described earlier, while normalization, the cost attribute of the ideal solution is converted into the benefit attribute. Thus, the optimal value of the cost attribute is written as 1 instead of 0. The experimental results represented in Table 12 demonstrate that for the 18th and 7th solutions, the order of rankings are in opposite according to the classical TOPSIS model, while two normalization procedures are involved. However, in this dataset, we observe that the 18th solution has 0 coverage (i.e., the first objective value) and it is ranked better than Solution 7, which has at least some good values in the coverage than Solution 18 according to TOPSIS (min-max normalization). Hence, if the traditional TOPSIS model with the positive ideal solution [1 1] is used, the perfect ordering of the two solutions remain ambiguous. Moreover, we cannot treat Solution 18 as a good solution according to this crowd judgment problem. On the other hand, the 18th solution is ranked higher than Solution 12 according to the traditional TOPSIS model, when min-max normalization procedure is considered. However, the contradictory results can be obtained, when the normalization procedure is changed with TOPSIS vector normalization. From the basic perception, it can be noticed that the 18th solution cannot be a good solution and these anomalies can be removed according to the proposed ideal solution obtained from the multi-objective approach. Moreover, we cannot rely on the single ranking, rather an aggregation of multiple rankings is performed and finally crowd workers are ranked according to the aggregated ranking. In this aggregated ranking also, the above mentioned ambiguities are resolved. We first apply the aggregated ranking algorithm considering all the individual rankings obtained from TOPSIS. We also find the aggregated ranking considering only the first one among three individual proposed ranking and these results also show the effectiveness of the proposed work. To illustrate, Solution 12 is ranked better than Solution 18 in both the aggregated rankings. Again, Solution 7 is ranked better than Solution 18 in the aggregated ranking. Therefore, the effectiveness of the proposed model is realized clearly.

To compare the performance for this second dataset with other state-of-the-art approaches as mentioned in [20,21], we perform another set of extensive analysis and the results are reported in Table 13. The ranking proposed by [21] (termed as Weighted TOPSIS), imposes weight 0.9171 for the first objective and 0.0829 for the second objective. Therefore, the ranking is mainly performed based on the first objective neglecting the second objective. It can be noticed from Table 13, Solution 9 has a very closer value with Solution 15 in terms of the first objective values. Although, Solution 15 has very poor value than Solution 9 in terms of second objective values, still, Solution 15 is ranked in higher position than Solution 9. Solution 9 has the 3rd lowest objective value in terms of the second objective, thus should be ranked better than Solution 15 if the weight on the both of the conflicting objectives could have been performed in a better way. Similarly, for Solution 3 and Solution 15, both the solutions have very closer values in terms of the first objective. But Solution 3 has a much lower value in terms of the second objective than Solution 15, so Solution 3 should have better ranked than Solution 15. However, it can be noticed that Solution 15 is ranked as better than Solution 3 that reveals the ineffability of this particular approach. Similarly, the another method PE-TOPSIS mentioned in [20] applies 0.6669 as a weight for the first objective function, 0.3301 weight for the second objective function. It can be seen that Solution 12 is ranked a high position, that is, Position 5 as the higher weight (double) is kept on the first objective function. It can be noticed that Solution 12 has a very poor second objective value, but as this method sorts the solutions mainly on the first objective values, so it is ranked higher. It can be seen that Solutions 1, 4, 14, and 11 have good values in terms of both the objectives, so they are ranked in the better positions. But Solution 12 has the second largest second objective value so it should not be ranked in the 5th position if both the objectives are considered. However, the proposed approach keeps Solution 12 as the 8th position as it makes a better trade-off between both the objectives. The ranking obtained by PSD-TOPSIS [20] applies 0.7306 weight as the first objective and 0.2694 as the weight for the second objective. The solutions obtained by the proposed method assigns Solution 3 as higher ranked than Solution 9, as more weight is imposed on the first objective value. However, it can be easily noticed that, Solution 3 and Solution 9 have very similar first objective values, whereas, the second objective function value is much better (2nd lowest) for Solution 9. Thus, Solution 9 should have been ranked better if both the objective functions are considered appropriately. The proposed approach removes these anomalies as it finds the weight in a more appropriate manner by optimizing the two conflicting objective functions simultaneously. To perform more experiments, we again consider the only one filtered solution instead of the average values of all the filtered solutions in order to design the ideal solution. In these proposed ideal solutions, that is, [1 0.6244], [1 1], and [1 0.7699] (as demonstrated in Table 14), the similar kind of the ranking is obtained in majority cases. In these ranking, the positions of Solutions 18 and 12 are resolved. All the proposed ideal solutions place Solution 18 as Positions 11, 10, and 8, respectively. Thus, the effectiveness of the proposed method in order to produce the better ranking utilizing the proper ideal solution is demonstrated.

## 6. Conclusions

In this paper, our main objective is to leverage the vast human resources of the crowd to solve different decision making problems for urban planning. In particular, the objective of this work is to find a ranking of the crowd, while the problem setting is formulated in the constrained judgment analysis framework. In this constrained judgment setting, due to the unavailability of the defined option set, it becomes very difficult to apply the traditional judgment analysis algorithms and thus obtaining the ranking of the crowd becomes complex. Even the simple majority voting is not applicable due to the presence of multiple components in a single question. Thus, to mitigate the problem, a multi-objective evolutionary approach is proposed to find a better ideal solution from the crowd while making a trade-off between conflicting objectives. Then we utilize that solution in order to define the appropriate positive ideal solution of a widely adopted group decision making tool, namely, TOPSIS. Thus, the experimental results over the two crowdsourced datasets for urban planning demonstrate the effectiveness of the proposed model.

In the future, the investigation can be done for other kinds of constrained crowd judgment analysis problems, where the opinions can be not necessarily numeric, rather categorical attributes can be present there. Furthermore, in this current context, the opinions from the crowd are collected in a single phase. However, to ensure better quality, the opinion from the crowd will be collected in the second phase and the ideal solution can be constructed from the two sets of solutions. The problem of collecting two-phase opinions inherently needs to obtain a ranking among the crowd in order to motivate the crowd to cast their opinions for the second time. Therefore, there is enormous scope for further improvement of this research. In this problem, the crowd workers provide the opinions, while they do not judge others’ opinions. So, further research can be investigated if instead of providing only one response, the crowd workers can provide their own responses while judging others’ opinions. Thus automatically, some opinions can be judged by the crowd workers at the same time of collecting opinions. However, there are many challenges on how to select which response from multiple responses should be provided to the crowd judged by the crowd workers. The reason is that one crowd worker cannot provide judgment for multiple responses, hence whether these questions are to be displayed to a specific crowd worker is also an important direction. Thus, there are multiple avenues for performing further research in this new type of a judgment analysis model.

## Figures and Tables

**Table 1:** Performance for the top-10 solutions (according to the first objective) of original crowd for the first dataset.

Solutions	Objective 1	Objective 2
Solution 1	1.2000	0.0499
Solution 2	1.1375	0.0551
Solution 3	0.9625	0.0509
Solution 4	0.5750	0.0326
Solution 5	0.5580	0.0395
Solution 6	0.4625	0.0441
Solution 7	0.4500	0.0258
Solution 8	0.3875	0.0446
Solution 9	0.3750	0.0346
Solution 10	0.3375	0.0393

**Table 2:** Performance measure for the top-10 solutions (according to the first objective) evolved after applying the proposed algorithm for the first dataset. Here, population size = 80 and generation number = 50.

Solutions	Objective 1	Objective 2
Solution 1	1.6890	0.0600
Solution 2	1.6631	0.0570
Solution 3	1.6325	0.0420
Solution 4	1.6299	0.0420
Solution 5	1.6164	0.0494
Solution 6	1.5809	0.0422
Solution 7	1.5688	0.0586
Solution 8	1.5536	0.0407
Solution 9	1.5536	0.0407
Solution 10	1.5535	0.0570

**Table 3:** Performance measure for the top-10 solutions (according to the first objective) evolved after applying the the proposed algorithm for the first dataset. Here, population size = 100 and generation number = 60.

Solutions	Objective 1	Objective 2
Solution 1	2.0476	0.0583
Solution 2	1.9723	0.0864
Solution 3	1.9660	0.0611
Solution 4	1.9414	0.0608
Solution 5	1.9384	0.0579
Solution 6	1.9349	0.0572
Solution 7	1.9348	0.0633
Solution 8	1.9240	0.0605
Solution 9	1.9110	0.0567
Solution 10	1.8188	0.0561

**Table 4:** Solutions obtained after filtering based on a reference solution for the first dataset. The filtered solutions are obtained for Population size = 100, generation number = 60.

Solutions	Objective 1	Objective 2
Solution 1	1.4636	0.0392
Solution 2	1.5140	0.0399
Solution 3	1.5920	0.0410
Solution 4	1.7096	0.0475
Solution 5	1.6028	0.0413
Solution 6	1.2414	0.0354
Solution 7	1.5171	0.0409
Solution 8	1.4496	0.0390
Solution 9	1.3386	0.0370
Solution 10	1.4103	0.0375
Solution 11	1.2000	0.0499

**Table 5:** Performance analysis of different rankings including TOPSIS (min-max normalization and vector normalization) and using the proposed ideal solutions for the first dataset.

	Objective 1	Objective 2	TOPSIS Min-Max + Ve Ideal = [1 1] − Ve Ideal = [0 0]	TOPSIS Vector Normalization + Ve Ideal = [1 1] − Ve Ideal = [0 0]	MOGA (1st Execution) + Ve Ideal = [1 0.3558] − Ve Ideal = [ 0 0]	MOGA (2nd Execution) + Ve Ideal = [1 0.4590] − Ve Ideal = [ 0 0]	MOGA (3rd Execution) + Ve Ideal = [1 0.3781] − Ve Ideal = [ 0 0]	Aggregated Ranking (Considering All MOGA Ranking)	Aggregated Ranking (Considering Only One MOGA Ranking)
Solution 1	0.9625	0.0509	6	4	3	3	3	3	3
Solution 2	0.1000	0.0363	15	17	17	17	17	17	17
Solution 3	0.3750	0.0346	8	8	8	8	8	8	8
Solution 4	0.1125	0.0402	19	19	19	19	19	19	19
Solution 5	0.4625	0.0441	11	11	10	10	10	10	10
Solution 6	0.3200	0.0592	20	20	20	20	20	20	20
Solution 7	0.1125	0.0344	12	14	14	14	14	14	13
Solution 8	0.4500	0.0258	2	5	5	5	5	5	4
Solution 9	0.2250	0.0423	18	18	18	18	18	18	18
Solution 10	0.1500	0.0355	14	16	15	15	15	15	15
Solution 11	0.5580	0.0395	7	7	6	6	6	6	7
Solution 12	0.5750	0.0326	3	6	4	4	4	4	6
Solution 13	0	0.0157	5	3	7	7	7	7	5
Solution 14	0.1500	0.0354	13	15	13	13	13	13	14
Solution 15	0.3875	0.0446	17	13	16	16	16	16	16
Solution 16	0.3375	0.0393	10	12	11	11	11	11	11
Solution 17	1.2000	0.0499	1	1	1	1	1	1	1
Solution 18	1.1375	0.0551	4	2	2	2	2	2	2
Solution 19	0.2250	0.0300	9	9	9	9	9	9	9
Solution 20	0.5000	0.0482	16	10	12	12	12	12	12

**Table 6:** Performance analysis of different rankings including Weighted TOPSIS, PE-TOPSIS and PSD-TOPSIS using the proposed ideal solutions for the first dataset.

	Objective 1	Objective 2	Weighted TOPSIS [21] + Ve Ideal = [0.45 0.0086] − Ve Ideal = [0 0.0324]	PE TOPSIS [20] + Ve Ideal = [0.2317 0.0465] − Ve Ideal = [0 0.1748]	PSD TOPSIS [20] + Ve Ideal = [0.3625 0.0242] − Ve Ideal = [0 0.0910]
Solution 1	0.9625	0.0509	3	3	3
Solution 2	0.1000	0.0363	19	19	19
Solution 3	0.3750	0.0346	10	9	10
Solution 4	0.1125	0.0402	18	20	18
Solution 5	0.4625	0.0441	7	8	8
Solution 6	0.3200	0.0592	12	18	11
Solution 7	0.1125	0.0344	17	17	17
Solution 8	0.4500	0.0258	8	6	7
Solution 9	0.2250	0.0423	14	13	14
Solution 10	0.1500	0.0355	16	16	16
Solution 11	0.5580	0.0395	5	5	5
Solution 12	0.5750	0.0326	4	4	4
Solution 13	0	0.0157	20	14	20
Solution 14	0.1500	0.0354	15	15	15
Solution 15	0.3875	0.0446	9	10	9
Solution 16	0.3375	0.0393	11	11	11
Solution 17	1.2000	0.0499	1	1	1
Solution 18	1.1375	0.0551	2	2	2
Solution 19	0.2250	0.0300	13	12	13
Solution 20	0.5000	0.0482	6	7	6

**Table 7:** Performance analysis of different rankings including TOPSIS (min-max normalization and vector normalization) and using the proposed ideal solutions for the first dataset.

	Objective 1	Objective 2	TOPSIS Min-Max + Ve Ideal = [1 1] − Ve Ideal = [0 0]	TOPSIS Vector Normalization + Ve Ideal = [1 1] − Ve Ideal = [0 0]	MOGA (1st Execution) + Ve Ideal = [1 0.5569] − Ve Ideal = [0 0]	textbfMOGA (2nd Execution) + Ve Ideal = [1 0.5262] − Ve Ideal = [0 0]	MOGA (3rd Execution) + Ve Ideal = [1 0.5824] − Ve Ideal = [0 0]	Aggregated Ranking (Considering All MOGA Ranking)	Aggregated Ranking (Considering Only One MOGA Ranking)
Solution 1	0.9625	0.0509	6	4	3	3	3	3	3
Solution 2	0.1000	0.0363	15	17	17	17	17	17	17
Solution 3	0.3750	0.0346	8	8	7	7	8	8	8
Solution 4	0.1125	0.0402	19	19	19	19	19	19	19
Solution 5	0.4625	0.0441	11	11	10	10	10	10	10
Solution 6	0.3200	0.0591	20	20	20	20	20	20	20
Solution 7	0.1125	0.0344	12	14	16	15	14	15	14
Solution 8	0.4500	0.0258	2	5	5	5	5	5	4
Solution 9	0.2250	0.0423	18	18	18	18	18	18	18
Solution 10	0.1500	0.0355	14	16	15	16	15	16	16
Solution 11	0.5580	0.0395	7	7	6	6	6	6	7
Solution 12	0.5750	0.0326	3	6	4	4	4	4	6
Solution 13	0	0.0157	5	3	8	8	7	7	5
Solution 14	0.1500	0.0354	13	15	14	14	13	14	15
Solution 15	0.3875	0.0446	17	13	13	13	16	13	13
Solution 16	0.3375	0.0393	10	12	11	11	11	11	11
Solution 17	1.2000	0.0499	1	1	1	1	1	1	1
Solution 18	1.1370	0.0551	4	2	2	2	2	2	2
Solution 19	0.2250	0.0300	9	9	9	9	9	9	9
Solution 20	0.5000	0.0483	16	10	12	12	12	12	12

**Table 8:** Performance for the top-10 solutions of original crowd solutions for the second dataset.

Solutions	Objective 1	Objective 2
Solution 1	1.3305	0.0258
Solution 2	1.2290	0.0237
Solution 3	1.0375	0.0189
Solution 4	0.9780	0.0190
Solution 5	0.8250	0.0346
Solution 6	0.5905	0.0198
Solution 7	0.5575	0.0336
Solution 8	0.5560	0.0284
Solution 9	0.5500	0.0194
Solution 10	0.5250	0.0297

**Table 9:** Performance for the top-10 solutions (according to the first objective) evolved after applying the proposed algorithm for the second dataset. Here, population size = 80 and generation number = 50.

Solutions	Objective 1	Objective 2
Solution 1	1.5258	0.0412
Solution 2	1.5140	0.0391
Solution 3	1.5122	0.0327
Solution 4	1.4674	0.0238
Solution 5	1.4672	0.0229
Solution 6	1.4539	0.0244
Solution 7	1.4525	0.0235
Solution 8	1.4358	0.0252
Solution 9	1.4175	0.0375
Solution 10	1.4097	0.0241

**Table 10:** Performance for the top-10 solutions (according to the first objective) evolved after applying the proposed algorithm for the second dataset. Here, population size = 100 and generation number = 60.

Solutions	Objective 1	Objective 2
Solution 1	1.9359	0.0421
Solution 2	1.9271	0.0397
Solution 3	1.8708	0.0394
Solution 4	1.8646	0.0405
Solution 5	1.8636	0.0391
Solution 6	1.8313	0.0414
Solution 7	1.7939	0.0384
Solution 8	1.7286	0.0397
Solution 9	1.7035	0.0297
Solution 10	1.7013	0.0384

**Table 11:** Solutions obtained after filtering based on a reference solution for the second dataset. The filtered solutions are obtained for population size = 100 and generation number = 60.

Solutions	Objective 1	Objective 2
Solution 1	1.3915	0.0202
Solution 2	1.6282	0.0251
Solution 3	1.3594	0.0203
Solution 4	1.3665	0.0227
Solution 5	1.3305	0.0257

**Table 12:** Performance analysis of different rankings including TOPSIS (min-max normalization and vector normalization) and using the proposed ideal solutions for the second dataset.

	Objective 1	Objective 2	TOPSIS Min-Max + Ve Ideal = [1 1] − Ve Ideal = [0 0]	TOPSIS Vector Normalization + Ve Ideal = [1 1] − Ve Ideal = [0 0]	MOGA (1st Execution) + Ve Ideal = [1 0.7318] − Ve Ideal = [ 0 0]	MOGA (2nd Execution) + Ve Ideal = [1 0.7582] − Ve Ideal = [0 0]	MOGA (3rd Execution) + Ve Ideal = [1 0.7699] − Ve Ideal = [0 0]	Aggregated Ranking (Considering All MOGA Ranking)	Aggregated Ranking (Considering Only One MOGA Ranking)
Solution 1	1.2290	0.0237	3	2	2	1	2	1	2
Solution 2	0.5905	0.0198	5	5	5	5	5	5	5
Solution 3	0.5560	0.0283	7	8	7	7	7	7	7
Solution 4	1.3305	0.0258	4	1	1	2	1	2	1
Solution 5	0.4495	0.0295	11	12	11	11	11	11	11
Solution 6	0.2945	0.0338	17	16	17	17	17	17	17
Solution 7	0.5250	0.0297	10	9	9	9	9	9	9
Solution 8	0.0100	0.0302	16	18	16	16	16	16	16
Solution 9	0.5500	0.0194	6	6	6	6	6	6	6
Solution 10	0.1725	0.0286	12	15	14	14	14	14	14
Solution 11	0.9780	0.0190	2	4	4	4	4	4	4
Solution 12	0.8250	0.0346	9	7	8	8	8	8	8
Solution 13	0.3580	0.0370	18	17	18	18	18	18	18
Solution 14	1.0375	0.0189	1	3	3	3	3	3	3
Solution 15	0.5575	0.0336	13	10	13	13	13	13	13
Solution 16	0.5125	0.0323	12	11	12	12	12	12	12
Solution 17	0.3630	0.0309	14	14	15	15	15	15	15
Solution 18	0	0.0239	8	13	10	10	10	10	10

**Table 13:** Performance analysis of different rankings including Weighted TOPSIS, PE-TOPSIS and PSD-TOPSIS using the proposed ideal solutions for the second dataset.

	Objective 1	Objective 2	Weighted TOPSIS [21] + Ve Ideal = [0.4125 0.0131] − Ve Ideal = [0 0.0256]	PE TOPSIS [20] + Ve Ideal = [0.3079 0.0521] − Ve Ideal = [0 0.1019]	PSD TOPSIS [20] + Ve Ideal = [0.3358 0.0425] − Ve Ideal = [0 0.0832]
Solution 1	1.2290	0.0237	2	2	2
Solution 2	0.5905	0.0198	6	6	6
Solution 3	0.5560	0.0283	8	8	7
Solution 4	1.3305	0.0258	1	1	1
Solution 5	0.4495	0.0295	12	12	12
Solution 6	0.2945	0.0338	15	15	15
Solution 7	0.5250	0.0297	10	10	10
Solution 8	0.0100	0.0302	18	17	17
Solution 9	0.5500	0.0194	9	7	8
Solution 10	0.1725	0.0286	16	16	16
Solution 11	0.9780	0.0190	4	4	4
Solution 12	0.8250	0.0346	5	5	5
Solution 13	0.3580	0.0370	14	14	14
Solution 14	1.0375	0.0189	3	3	3
Solution 15	0.5575	0.0336	7	9	9
Solution 16	0.5125	0.0323	11	11	11
Solution 17	0.3630	0.0309	13	13	13
Solution 18	0	0.0239	12	18	18

**Table 14:** Performance analysis of different rankings including TOPSIS (min-max normalization and vector normalization) and using the proposed ideal solutions for the second dataset.

	Objective 1	Objective 2	TOPSIS Min-Max + Ve Ideal = [1 1] − Ve Ideal = [0 0]	TOPSIS Vector Normalization + Ve Ideal = [1 1] − Ve Ideal = [0 0]	MOGA (1st Execution) + Ve Ideal = [1 0.6244] − Ve Ideal = [ 0 0]	MOGA (2nd Eexecution) + Ve Ideal = [1 1] − Ve Ideal = [ 0 0]	MOGA (3rd Execution) + Ve Ideal = [1 0.7676] − Ve Ideal = [0 0]	Aggregated Ranking (Considering all MOGA Rankings)
Solution 1	1.2290	0.0237	3	2	2	3	1	1
Solution 2	0.5905	0.0198	5	5	5	5	5	5
Solution 3	0.5560	0.0284	7	8	7	7	7	7
Solution 4	1.3300	0.0258	4	1	1	4	2	2
Solution 5	0.4495	0.0296	11	12	10	11	11	11
Solution 6	0.2945	0.0338	17	16	17	17	17	17
Solution 7	0.5250	0.0297	10	9	9	10	9	9
Solution 8	0.0100	0.0302	16	18	16	16	16	16
Solution 9	0.5500	0.0194	6	6	6	6	6	6
Solution 10	0.1725	0.0286	12	15	15	13	13	13
Solution 11	0.9780	0.0190	2	4	4	2	4	4
Solution 12	0.8250	0.0346	9	7	8	9	8	8
Solution 13	0.3580	0.0370	18	17	18	18	18	18
Solution 14	1.0375	0.0189	1	3	3	1	3	3
Solution 15	0.5575	0.0336	13	10	13	14	14	14
Solution 16	0.5125	0.0323	12	11	12	12	12	12
Solution 17	0.3630	0.0309	14	14	14	15	15	15
Solution 18	0	0.0239	8	13	11	8	10	8

## Data Availability

Not applicable.
